# Electronic medical records in humanitarian emergencies – the development of an Ebola clinical information and patient management system

**DOI:** 10.12688/f1000research.8287.3

**Published:** 2017-02-23

**Authors:** Kiran Jobanputra, Jane Greig, Ganesh Shankar, Eric Perakslis, Ronald Kremer, Jay Achar, Ivan Gayton

**Affiliations:** 1 Manson Unit, Médecins sans Frontières (MSF), London, UK; 2 Google Crisis Response Team, Mountain View, CA, USA; 3 Centre for Biomedical Informatics and Department of Global Health and Social Medicine, Harvard Medical School, Harvard University, Boston, MA, USA; 4 MSF, Amsterdam, Netherlands

**Keywords:** Ebola, electronic medical records, EMR, innovation, humanitarian

## Abstract

By November 2015, the West Africa Ebola epidemic had caused 28598 infections and 11299 deaths in the three countries most affected. The outbreak required rapid innovation and adaptation. Médecins sans Frontières (MSF) scaled up its usual 20-30 bed Ebola management centres (EMCs) to 100-300 beds with over 300 workers in some settings. This brought challenges in patient and clinical data management resulting from the difficulties of working safely with high numbers of Ebola patients. We describe a project MSF established with software developers and the Google Social Impact Team to develop context-adapted tools to address the challenges of recording Ebola clinical information. We share the outcomes and key lessons learned in innovating rapidly under pressure in difficult environmental conditions. Information on adoption, maintenance, and data quality was gathered through review of project documentation, discussions with field staff and key project stakeholders, and analysis of tablet data. In March 2015, a full prototype was deployed in Magburaka EMC, Sierra Leone. Inpatient data were captured on 204 clinical interactions with 34 patients from 5 March until 10 April 2015. Data continued to also be recorded on paper charts, creating theoretically identical record “pairs” on paper and tablet. 83 record pairs for 33 patients with 22 data items (temperature and symptoms) per pair were analysed. The overall Kappa coefficient for agreement between sources was 0.62, but reduced to 0.59 when rare bleeding symptoms were excluded, indicating moderate to good agreement. The time taken to deliver the product was more than that anticipated by MSF (7 months versus 6 weeks). Deployment of the tablet coincided with a dramatic drop in patient numbers and thus had little impact on patient care. We have identified lessons specific to humanitarian-technology collaborative projects and propose a framework for emergency humanitarian innovation. Time and effort is required to bridge differences in organisational culture between the technology and humanitarian worlds. This investment is essential for establishing a shared vision on deliverables, urgency, and ownership of product.

## Background

By November 2015, the West Africa Ebola epidemic had caused 28598 infections and 11299 deaths in the three countries most affected ^1^. In response, Médecins sans Frontières (MSF), drawing on over 20 years of experience in responding to Ebola outbreaks, had treated more than 7500 suspected Ebola cases, including more than 4700 confirmed cases. The outbreak, unprecedented in size, required rapid innovation and adaptation. To cope with the number of cases, MSF scaled up its usual 20–30 bed Ebola management centres (EMCs) to 100–300 beds with over 300 workers in some settings. This increased scale brought challenges in patient and clinical data management resulting from the difficulties of working safely with high numbers of Ebola patients ( Panel 1). Little had been published on managing clinical documentation and data transfer from filovirus wards, and there were no established, standardized, or widely used approaches ^2^. Here we describe how MSF developed context-adapted tools to address the challenges of recording Ebola clinical information for in-patient management. We share the key issues and lessons learned in innovating rapidly under pressure in difficult environmental conditions and propose a framework for emergency humanitarian innovation.

**Panel 1.** Challenges in patient and clinical data management in Ebola management centres.

• Staff working in the high-risk zone of an EMC (the area where there is high risk of Ebola infection) must wear PPE, which is heavy, limits vision severely, adversely affects manual dexterity, and is extremely hot. Clinicians have a strictly limited time in which they can safely remain in PPE without overheating and increasing infection risk during PPE removal. A lot of time was lost while clinicians in PPE tried to find patients inside the high risk zone of the EMC. Patients were moved through tents in the EMC representing different levels of risk during their treatment and could also move around within the EMC of their own volition (patients can become confused and delirious during Ebola infection), which made locating a specific patient in a large EMC challenging. • Clinicians lacked an efficient means to document patient history and record patients’ progress through the EMC. In some EMCs, clinicians in full PPE shouted data over the fence for someone outside the high-risk zone to record on ‘clean’ paper, or data were recorded in digital photographs, since nothing could be taken out of the high-risk zone without being disinfected in chlorine. The original paper records made in the high-risk zone were incinerated. As a result, adequate data to support good clinical care were rarely available. • Data collection processes varied widely between EMCs, resulting in little data sharing between them and limiting the potential for learning and improving care. There was no centralized organizing authority for data collection forms or formats. • Despite the efforts of the Emergency Telecommunications Cluster *, most EMCs had spotty and intermittent internet connectivity at best.

EMC=Ebola management centre. PPE=personal protective equipment.

*Information on the ETC is at: http://www.etcluster.org/about-etc

## Methods

### The project

In September 2014, MSF medical staff established a collaborative project with a small group of software developers and the Google Social Impact Team. The aim was to develop a tool to enable the collection, visualisation, and sharing of standardised information on Ebola patients and treatment programmes, and thereby make the most efficient use of the limited time that staff are able to remain in personal protective equipment (PPE) to treat patients in the high-risk zone of an EMC.

Informal discussions with current and returning MSF field staff enabled a first set of requirements to be drawn up. Initial scoping, conducted by Google over 2 days, showed that several data collection devices were already under development for Ebola, some of which promised to be rapidly deployable ^3, 4^. However, it was determined that none of these would sufficiently meet the requirements that had been identified. These requirements were further refined through extensive user consultation including current MSF national and international field staff, operations managers, and medical specialists with experience in Ebola, as well as public health specialists from Harvard Medical School and The London School of Hygiene and Tropical Medicine. A product specification was generated to meet these requirements ( Panel 2) based on the following principles: the solution should be available quickly (within 6 weeks); it should be “just enough” to meet the requirements; it should be useable without programming knowledge; and both the product and its intellectual property (IP) should be freely or cheaply available.

**Panel 2.** Core requirements and the initial proposed solution (the minimum viable product).

Requirements	Solution (minimum viable product)
Clinicians within the high-risk zone wearing PPE are able to quickly, easily, and safely record and visualise patient data, to enable appropriate clinical decision making.	Waterproof tablets with wireless charging, disinfected by dipping in 0.5% chlorine. A tablet app enabling rapid data entry in PPE (large buttons, no swipe-screen features), providing instant access to patient historical records, a current patient list which staff can fill during clinical rounds, and an interface for the creation of more fields as required.
Data transfer out of the high-risk zone is easy, safe, and secure, enabling aggregation and analysis of outcomes (programme monitoring).	A local server-side application to synchronise data between tablets, store it securely on a server outside the high-risk zone, and export anonymised patient data (to the field office and headquarters in Europe) when an internet connection is available.
System functions with unreliable power supply (up to 12 hours without electricity), poor or no internet connectivity, and no in-country IT support desk or readily available electronics supply.	Lightweight, plug-and-play, battery-powered server infrastructure, automatically backed up on every device in case some fail.
Clinicians within the high-risk zone able to locate patients quickly, easily, and safely.	Radio-frequency identification tags on wristbands to rapidly locate patients within the centre and confirm their identity; readers for the tags located on each tablet device.

PPE=personal protective equipment.

To speed up development and ensure that the final tools would be available free of charge to Ministries of Health or other humanitarian organisations, the development team used pre-existing open-source platforms where possible, rather than developing from scratch. The data model and database from OpenMRS (an open-source Medical Record System; platform v 1.10.1 on an Edison server running Yocto Linux + Debian GNU/Linux 7) were combined with data entry elements from OpenDataKit (open-source mobile data collection tools), together with a bespoke user interface (made with code forked from ODK Collect v1.4.4, running on Android 4.4.2 [KitKat]), designed for a high-risk zone. Panel 3 outlines the tool that was finally developed. A full, open-source, technical specification of the product is available ^5^.

**Panel 3.** Core components of the final Ebola data management solution.

**Hardware** Sony Android tablets (Sony Experia Z2 running Android 4.4.2 [KitKat]) in a custom-built polycarbonate casing containing a radio- frequency identification reader and rechargeable batteries, custom-built by Thinkify. The server (Edison server running Yocto Linux + Debian GNU/Linux) and local area network were based on a federated Edison server structure (housed in individual waterproof polycarbonate boxes developed by Thinkify) powered by rechargeable batteries linked to mains electricity. **Software** The tablet software is a custom-built Android app. The server-side application (back end) is OpenMRS (platform v 1.10.1) built on a Linux Operating System running on a low-energy server (Intel Edison), with a custom-built module for data import from the Android app (imported data is anonymised but linkable to an individual if required, using a patient ID code and approximate date of birth based on current age). **Data content** The following data fields were hard-coded: • Identifiers: patient identification number, approximate date of birth, encounter time and date, date of EMC admission. • Bleeding: blood in stool (black/red), red eyes, bleeding from eyes, nosebleed, haemoptysis, haematemesis, vaginal bleeding, haematuria, oral bleeding, bleeding NOS. • Other symptoms: chest pain, muscle/joint pain, difficulty breathing, hiccups, heartburn, headache, cough, back pain, sore throat, abdominal pain (upper/lower), nausea. • Signs: consciousness, hydration, diarrhoea (episodes/24 hours), mobility, vomiting (episodes/24 hours), blood pressure, heart rate, temperature (°C), weight (kg), weakness, respiratory rate, pregnant, diet, IV access present, pain level. • Laboratory test results: Ebola NP gene PCR CT value, Ebola L gene PCR CT value.

EMC=Ebola management centre. NOS=not otherwise specified. IV=intravenous.

The client-server architecture developed for this project involves a low-energy server implemented on a 36 × 25 × 4 mm Intel Edison computer, built into an enclosure full of batteries and a custom charging circuit to ensure all-hours reliability. Rather than relying on the internet for updates, backups, and maintenance, the backup and updating system relies solely on USB sticks. The client software installed on the tablets can be replaced with any other application, which can then benefit from the hardware and server capacity of the system. Servers can be deployed inside and outside the high risk zone, enabling safe and effortless transfer of data between locations.

Approximately US$1.9 million was spent on development, of which $1.8 million was provided by Google. This included a team of five full-time engineers for 6 months, as well as contractors and consultants. Approximately $500,000 was spent on custom manufacturing of hardware, often at above-market costs due to the urgency of the project. For instance, a factory in California was commissioned to run for 72 hours straight during the Christmas of 2014 to manufacture tablet enclosures. In all, the project took 7 months from concept to deployment.

### Piloting and evaluation

In January 2015, an alpha version was field-piloted in the MSF EMC in Magburaka, Sierra Leone; feedback from the field team was incorporated and bugs were fixed. In March 2015, a full prototype was deployed in Magburaka. Inpatient data were captured on 204 clinical interactions with 34 patients from 5 March until 10 April 2015 (equating to 95% of those admitted in this relatively quiet period). In this initial deployment, for each clinician-patient interaction the routine paper record system was maintained in parallel; clinical observations and treatments were recorded on paper in the high risk zone by the clinician wearing PPE, then shouted over the fence to a colleague standing in the low-risk zone (an area of an EMC where there is low risk of Ebola transmission and staff do not wear full PPE) who transcribed them to ‘clean’ paper patient charts, and later entered into an Excel database by a data encoder.

Information on adoption, maintenance, and data quality was gathered through review of project documentation, discussions with field staff and key project stakeholders, and analysis of data from the tablets. We carried out a rapid informal mixed methods evaluation to look at adoption/acceptance by health workers, implementation and maintenance challenges, and data quality and usefulness. Observation of staff and six unstructured staff interviews were carried out by a member of the implementation team with experience of implementing and evaluating e-health innovations, and were recorded in note form. Six semi-structured interviews with key project stakeholders (Google, MSF, Harvard) were carried out using a topic guide by an MSF administrator with experience in qualitative research, who recorded and transcribed the interviews. Project documentation from September 2014 to April 2015, including situation reports, vision and requirements documents, were included as an additional data source. Thematic analysis of project documentation, observations and field interview notes, and stakeholder interview transcripts was performed by the administrator supported by a senior team member with experience in health-care evaluation.

Data from the tablets were analysed via a semi-automated match of record “pairs” (clinician-patient interactions where a record for the same interaction existed in both the tablet dataset and the paper→Excel dataset). For data items in both tablet and Excel datasets (temperature and symptoms), the simple data (raw data rather than OpenMRS codes) were extracted from the tablet dataset and multiple records for interactions within 30 minutes were manually merged. If a symptom was recorded as present in any record in a set of multiple records within 30 minutes, the symptom was marked as present. The Excel dataset was checked and corrected against scanned paper records. The aligned tablet and cleaned Excel datasets were combined into a single list, sorted by patient and date/time of interaction, such that the single daily record in Excel was paired with the first tablet record that day. This is a potential limitation, as it assumes that no observations would be recorded on the tablet prior to the principle morning clinical encounter, at which the single daily paper record was made. The record pairs were then recoded to collapse graded symptoms in the tablet record (e.g. 24 hour count of diarrhoea or vomiting episodes, extent of weakness) to only symptom present or absent, and some symptoms combined into a single symptom variable (e.g. abdominal pain was a single variable on paper but recorded separately for upper and lower on the tablet, so a combined variable was used for comparison). Temperature was recoded to <37.5°C or >=37.5°C, and also matched using precise value. Kappa coefficients were calculated using STATA v14.2 (StataCorp, College Station, Texas USA) for each symptom and for all records with an entry in both sources. Observations of vital signs were not part of the Excel dataset, but the presence of these items in the scanned paper records was documented and compared to whether these signs were also recorded in the tablet record. The quality of the scanned paper charts was subjectively assessed.

### Ethics

This evaluation met the criteria of the MSF Ethics Review Board for exemption from ethics review. The product was discussed with and demonstrated to health authorities prior to implementation, who were supportive of this innovation and did not suggest additional ethical scrutiny.

### Results/outcomes

The time taken to deliver the product was substantially more than that anticipated by MSF (7 months, as opposed to 6 weeks). The initial specification was largely but not fully delivered; a patient localising feature (radio-frequency identification tags for patients and a network of readers) was developed but never completed, as the declining patient numbers reduced the potential value of this feature. In addition, exported data require significant work to clean and analyse outside of the application interface. The database was not configured to create a single record for a patient encounter if the user moved in and out of the record, resulting in up to five partial records within a 10 minute period which all related to a single encounter, and thus required to be merged into a single record of that encounter. Records were then exported with content and order based on OpenMRS coding, resulting in 3 data fields per data element and an order that was non-intuitive and would require further development for the product to be usable in field conditions.

Due to the hasty decommissioning of the Google team at the end of the project, there was insufficient time for a comprehensive handover (from Google to MSF) of information and understanding required to operate and maintain the software and hardware that had been developed. In the case of the software and key hardware, MSF was able to hire an ex-Google engineer who worked on this project to further develop the software and complete the process of handover to MSF. However some parts of the hardware (e.g. polycarbonate casing) were deemed too expensive to warrant further investment by MSF, so little attempt was made to obtain the knowledge necessary to produce these.

Deployment of the tablet coincided with a dramatic drop in patient numbers. As a result, the full prototype had little impact on patient care.

***Adoption.*** The final product deployed in Magburaka was well received by medical staff, some of whom had no previous experience with computers or touch-screen devices. Staff described the system as intuitive and reliable for data entry and visualisation, even when power and internet connections were interrupted. Medical staff valued the ability to access more detailed, real-time, clinical data.

***Implementation and maintenance.*** Tablets remained functional after frequent dipping in chlorine disinfectant, and the system remained active for periods of up to 24 hours without electricity.

***Data quality and usefulness.*** 83 record “pairs” for 33 patients with 22 data items (temperature and symptoms) per pair were available to be analysed.

- The overall Kappa coefficient for agreement between both sources was 0.62, but reduced to 0.59 when rare bleeding symptoms were excluded, indicating moderate to good agreement. For individual symptoms it the Kappa coefficient ranged from 0.29 to 0.86, with chest pain, diarrhoea and sore throat having only fair agreement, but precise temperature very good agreement. The number of pairs with data available in both sources per symptom ranged from 78–81 of the 83 record pairs. Most patients were admitted for a short period and symptoms were recorded on paper only once per day, so 30/33 (91%) had 1–3 record pairs. One patient had 19 record pairs, but if all record pairs beyond 4 were excluded, the Kappa coefficient was still 0.59.
- The tablet contained more unique patient encounters than the paper records – the paper chart usually showed one set of observations per day, while the tablet was used to record additional encounters that were (at best) otherwise written only in patient progress notes (which were not standardised and thus difficult for a data encoder to enter into a standardised database format). However, it is unknown if this recording would still occur if the EMC were very busy.
- The tablet contained data fields for vital signs (BP, HR, RR) that were not always recorded on the paper vital signs charts, but rather only on the free-text clinician progress notes (which had not been entered into the Excel database at the time of matching). At least one of these vital signs was entered in the tablet for about 40% of 204 interactions, usually matching the paper record, and sometimes providing data that did not exist in the paper record. In a few instances (11/85 interaction pairs), one or more vital signs in the paper record were not in the tablet dataset.
- The paper charts were sometimes hard to read, leading to errors in the Excel database such as a temperature recorded in the paper record that could be read as either 39.2 or 34.2 (the tablet record showed 34.2). In addition, there was variable use in the symptoms chart of symbols such as yes, y, no, n, ✓, ✘, -, |, ?. About 20% of paper-recorded encounters had some ambiguity, including no or only partial zero reporting (that is, specifying that symptoms were absent, rather than an assumption that any symptom not marked as present is absent as opposed to not assessed).

Interviews with field staff and key stakeholders revealed these common themes around challenges faced in the project.

- *Lack of agreed vision from start*: The team’s haste to deploy something quickly that was “just enough” meant that insufficient time was taken to establish internal (MSF) project vision. Instead, the project team adopted a more perfection-driven Google vision (which extended the timeframe), and the user (MSF) questioned only late why something so complex had been made that was potentially useful in the long term, but not in this outbreak.
- *No explicit approach to project management*: MSF project management structure, including governance and an organogram, was defined only when the project started to falter. As a result, the software developers were never sure who was the MSF lead, or who to go to when things went wrong, so at times asked the wrong question of the wrong person and lost focus on the minimum viable product requirements. At a late stage, efforts were made to develop an evaluation plan, but the numbers of cases dropped very substantially soon after.
- *Business pressures*: the pressure to start meant that reflection on team composition happened too late. For example, the team approached numerous medical people with different backgrounds, experiences, and expectations for user input. Since the epidemic evolved rapidly, this focus on “the last medic returning” as the optimal source of information meant that the user stories became rapidly out of date. A consistent medical focal person was appointed to the team to provide ongoing guidance, but this was done too late. Likewise, Google’s need to move its developers onto other projects meant that there was insufficient time for effective knowledge transfer to a technologically-naïve partner.
- *Lack of understanding between the humanitarian and technology fields*: MSF expected to be able to describe what was needed quickly and then leave the software developers to deliver, without realising the time commitment required from them to enable the developers to make the right product. In parallel, the developers expected that health programmes could be measured in terms of number of patients accessed, and struggled to incorporate a health outcome perspective in their objectives and notion of impact.

## Discussion

The tablet facilitated more frequent and slightly more detailed data than the fence→ paper→Excel database routine, and there was high consistency between the results from the two systems. Given that the tablet data record is essentially real-time (in terms of data entry and opportunity for correction), it is likely to be the more accurate record. This assumption requires validation, but there is anecdotal evidence of the errors and uncertainties of the non-electronic data system from several EMCs.

To our knowledge, this is the first time a portable real-time electronic medical record (EMR) has been developed that specifically meets the needs of an extreme biohazard environment in a resource-limited setting with erratic power and internet supply. A client-server architecture normally necessitates a reliable, high-bandwidth internet connection, or a local server with very reliable electrical energy, IT support, and the availability of specialized parts. These requirements make a client-server architecture difficult to implement in rural sub-Saharan Africa.

MSF has gained experience and understanding of the process of technological innovation, including the limits and challenges of deploying new technology and working with technology partners ^6^. The positive collaboration between MSF and Google has sparked interest in the potential for humanitarian-technology collaborations ^7^. Successful deployment of reliable client-server architecture in a rural sub-Saharan African environment represents a proof of concept, such that it is already being deployed by other agencies (The Wellbody Alliance, in collaboration with Harvard Medical School).

However, there were major challenges with the project. Due to the length of time to deliver the product, in the context of an evolving Ebola outbreak, a product was delivered too late to have an impact on patient care or efficiency of EMC management. Lack of knowledge transfer meant that MSF had a product that they did not fully know how to support or adapt for future use.

The challenges we describe are echoed throughout the literature on humanitarian and technological innovation, such as best practice principles for humanitarian innovation defined by ALNAP and lessons learnt from IT innovation as outlined in the Chaos Manifesto ^8, 9^. Our experience has led us to identify some concrete lessons specific to humanitarian-technology collaborative projects. Most importantly, time and effort is required to bridge differences in organisational culture between the technology and humanitarian worlds. This investment is essential for establishing a shared vision on deliverables, urgency, and ownership of product. To guide this process, there is a need for a **framework for innovation** in humanitarian emergencies. The overwhelming priority for medical teams is immediate delivery of care, and innovation projects must fit around this reality; careful consideration must be given to whether there are sufficient resources to deliver the project. Therefore, emergency innovation projects need to be agile, iterative, and free from heavy project management processes. Yet if we are serious about innovation, we need to push the boundaries to ensure it has impact not only on our current patients, but also for future patients. We have outlined the key components of such a framework in Panel 4. This framework should be used as a flexible and practical tool; we caution against adapting systems that could lead to stifling of innovation, especially in complex and challenging environments where fast solutions are needed.

**Panel 4.** A framework for humanitarian-technology innovation projects

• Problem statement and project vision, with deliverables. • High-level requirements for the solution with early agreement on the minimum viable product. Users should be involved in defining requirements, and for each subsequent step. • Implementation requirements: clarifying who is involved and what they are responsible for; ensure they all have a common understanding of terms such as impact, and that the right parties are involved in decisions. • Implementation planning with timeline for key milestones (even if this is likely to evolve): planning, prototyping, piloting, roll out. Ensuring the time factored in for input and delivery is feasible. • Evaluation plan with expected impact and key performance indicators against which to measure success. • Communication and change management plan: how and who to handle internal updates and external communication; who needs to be consulted and who needs to be informed. • Governance: expert steering committee (or equivalent) which validates each of the above milestones; a mechanism for ethical oversight; clarity about who owns the IP that results.

An MSF team is in the process of adapting the software code-base of the Ebola data management solution to develop a generic emergency EMR that can be rapidly modified for different types of emergency. Our vision is to build a modifiable Open-MRS backbone for which new disease-specific apps can be developed within 5 days by a non-coder. The first ‘test-case’ is nutrition, for which we are working on apps for inpatient and ambulatory therapeutic feeding centres. In this project, having learnt from our experience with the Ebola EMR, we have taken the time to apply our framework ( Panel 4) from the start.

A complete kit of open-source software, hardware, and documentation for the Ebola EMR is available on-line and can be downloaded for free; the code is also freely available for developers to use ^5^. Several of the developers involved have formed an open-source community that is supporting this code, and can help develop the software for new-use cases ^10^. Harvard Medical School, MSF, and Google are all represented in this consortium. Harvard Medical School have carried out successful deployments of the hardware, which they have combined with new software for community surveillance, triage, and inpatient care. The consortium is focused upon new use-cases, the economics of the solution and driving economies of scale, the incorporation of additional software and hardware toolsets, and the enabling of clinical research during outbreak settings.

## Conclusion

The fundamental innovation of our project in the end, was not the new technology that was developed, but rather the adaptation of existing technology so that it would work in an environment that is generally hostile for complex systems. We hope that the lessons learned and the tools developed in this collaboration will help others involved in innovation in humanitarian crises to gain the balance between speed, impact, and sustainability.

## Data availability

The data referenced by this article are under copyright with the following copyright statement: Copyright: © 2017 Jobanputra K et al.

Data are deposited in a secure server in MSF Operational Centre Amsterdam and are available via a managed access process under the terms of the MSF Data Sharing Policy ^11^. For contact details and explanation of process please visit http://fieldresearch.msf.org/msf/handle/10144/306501 or email data.sharing@msf.org.
