# Supplementary material for: Electronic medical records in humanitarian emergencies – the development of an Ebola clinical information and patient management system
Source: F1000Res. 2017 Feb 23;5:1477. Originally published 2016 Jun 23. [Version 3] doi: 10.12688/f1000research.8287.3 (PMC5357025; doi:10.12688/f1000research.8287.3)
Supplement: Peer review history

# Peer review: Referee response for version 3

**DOI:** 10.5256/f1000research.11868.r20494

**Published:** 2017-03-10

**Authors:** James Whitworth, Hilary Bower

**Affiliations:** 1 Department of Infectious Disease Epidemiology, Faculty of Epidemiology and Population Health, London School of Hygiene and Tropical Medicine, London, UK; 2 Department of Infectious Disease Epidemiology, Faculty of Epidemiology and Population Health, London School of Hygiene & Tropical Medicine, London, UK

We thank the authors for their clarification of all the points that we have queried and for their revisions of the text. We are content to approve the paper fully now and congratulate the authors on an interesting contribution to the scientific literature.

 We note that the authors have used and interpreted the Kappa statistic as 'moderate to good' in the abstract and results, but called it 'high' consistency in the discussion. We think this apparent discrepancy is acceptable as for some elements, e.g. temperature the kappa statistic was very good. 

 We provide below a reference for the interpretation of the Kappa statistic for those who might require it. ^1^

We have read this submission. We believe that we have an appropriate level of expertise to confirm that it is of an acceptable scientific standard.

## References

[1] VieraAJGarrettJM: Understanding interobserver agreement: the kappa statistic. *Fam Med*.2005;37(5) :360-3 15883903

# Peer review: Referee response for version 2

**DOI:** 10.5256/f1000research.10435.r17187

**Published:** 2016-10-25

**Authors:** James Whitworth

**Affiliations:** 1 Department of Infectious Disease Epidemiology, Faculty of Epidemiology and Population Health, London School of Hygiene and Tropical Medicine, London, UK

Thank you for giving us the opportunity to review the revised article and explanations from the authors. In general they have responded and given clarifications either in the revised article, or the covering letter, and sometimes both. However, there are a few outstanding points that have not been addressed and we would like to see responses to these before we would be ready to amend the current status of approved with reservations.

 Our outstanding three comments are detailed below.

1. The authors have added a para about exporting data from the red zone, but it would still be good to have some simple text explaining exactly what staff needed to do to transfer the data, to help those readers who find ‘servers’ a technical term. We are still a bit puzzled about the reference to USB sticks for backup and updating and whether that refers only to outside the red zone back up, or internally.
2. As mentioned in our previous referee report:
*“The results are generally clearly described, and suggest that the tablet may be at least as good as paper records, though the analysis currently does not appear to take into account the role of chance, and a proportion of records (17% ?) had to be excluded. The qualitative study is quite weak. It appears that different data were collected systematically using the two different systems, so only some of the data were collected using both methods.*
*Further comments on the quantitative study are:*
*If we understand correctly the agreement between the two methods was calculated by simple percentage agreement: would it be possible to perform a Kappa test to take into account chance?*
*What was the range of interaction pairs per patient? Was any difference seen in the recording by either method related to the severity of the patient’s illness, or the number of people admitted at the time?*
*Is it possible to state the percentage of missing data overall and/or by recording method and comment on the implications for the agreement results? On rough calculation overall it seems to be 17% (204-(85x2) = 34, 34/204) missing data in one or other system.*
*The agreement between data sources ranged between 69 and 95%. This is rated as ‘high consistency’ but I was not sure on what basis. It was not clear to me, which collection method was felt to be the gold standard, or even if this was determined before the comparison. I sense from the discussion, the tablet was thought to perform better than the paper method, but I am not convinced about this from the results.”* ^1^
  These points are not addressed either in response or text – and the reference to high consistency remains (1 ^st^ para discussion)
3. Similarly, this point has also not been addressed:
*“The authors claim that this is the first such device to be developed. How did they search for other evidence of similar projects? I heard anecdotally of other projects, but am not aware whether they reached fruition, or have ever been published in any form. It would be good to have a thorough systematic search to establish what else has been developed.”* ^1^

I have read this submission. I believe that I have an appropriate level of expertise to confirm that it is of an acceptable scientific standard, however I have significant reservations, as outlined above.

## References

[1] BowerHWhitworthJ: Referee Report For:Electronic medical records in humanitarian emergencies – the development of an Ebola clinical information and patient management system [version 2; referees: 1 approved, 1 approved with reservations]. *F1000Research*.2016;5(1477) : 10.5256/f1000research.8913.r14552 10.5256/f1000research.8913.r14552 PMC535702528357037

# Response

**Published:** 2017-02-19

**Authors:** kiran jobanputra

**Affiliations:** Médecins sans Frontières , UK

The authors would like to thank the reviewer for the comments in their response letter, and have endeavoured to address the outstanding issues raised:

***Data transfer:*** *The authors have added a para about exporting data from the red zone, but it would still be good to have some simple text explaining exactly what staff needed to do to transfer the data, to help those readers who find ‘servers’ a technical term. We are still a bit puzzled about the reference to USB sticks for backup and updating and whether that refers only to outside the red zone back up, or internally.*

 We have addressed this in the text of the article (v3)

***Data accuracy:*** *If we understand correctly the agreement between the two methods was calculated by simple percentage agreement: would it be possible to perform a Kappa test to take into account chance?* *What was the range of interaction pairs per patient? Was any difference seen in the recording by either method related to the severity of the patient’s illness, or the number of people admitted at the time?* *Is it possible to state the percentage of missing data overall and/or by recording method and comment on the implications for the agreement results? On rough calculation overall it seems to be 17% (204-(85x2) = 34, 34/204) missing data in one or other system. The agreement between data sources ranged between 69 and 95%. This is rated as ‘high consistency’ but I was not sure on what basis. It was not clear to me, which collection method was felt to be the gold standard, or even if this was determined before the comparison. I sense from the discussion, the tablet was thought to perform better than the paper method, but I am not convinced about this from the results.”*

 The methods have been edited and percentage agreement results have been replaced with Kappa coefficients (v3). Data on missing results and the number of records per patient has been added.  Relatively few patients were admitted during the period, and bleeding symptoms were rare, so attempting to account for these issues is unlikely to be valid with this dataset. Neither method was a gold standard.

**Other comparable products:** *“The authors claim that this is the first such device to be developed. How did they search for other evidence of similar projects? I heard anecdotally of other projects, but am not aware whether they reached fruition, or have ever been published in any form. It would be good to have a thorough systematic search to establish what else has been developed.”* ^1^

 At the time of writing, we searched PubMed and Google using various search terms including “EMR”, “data collection” and “Ebola”, and found no description of similar products. Through our informal networks, we found out about two data collection devices that were developed for the ebola epidemic, but neither had EMR functionality. After submission of this article, we learnt about an ebola EMR developed by ThoughtWorks, but without the server infrastructure. Although a systematic review is beyond the scope of this article, we have add a reference to this other product in the text (v3).

# Peer review: Referee response for version 1

**DOI:** 10.5256/f1000research.8913.r15182

**Published:** 2016-08-23

**Authors:** Benjamin O. Black

**Affiliations:** 1 The Whittington Hospital, London, UK

Research and development of technology in the humanitarian arena is a rapidly growing field that encompasses many specialities. There are international working groups, peer reviewed journals and conferences dedicated to this field. This paper is a valuable addition to the body of evidence openly available to the academic, technician and aid worker. My review of this paper relies on my field experience of working in the humanitarian setting, particularly though the West African Ebola outbreak of 2014 till 2016.

 This paper describes the process, implementation and challenges of introducing a novel way of recording patient data electronically in a high biohazard environment. The aim being to improve data collection and the efficiency of patient interactions and communication.  

 It is bold and brave of the authors to openly state the errors made through the design and implementation of this project. While there is substantial discussion around the lessons identified from their experience, there could have also been more highlighting some of the possible successes, not least that this project was rolled out at a time of unprecedented pressures on the organisation responsible, Médecins Sans Frontières (MSF).

 In their peer review Whitworth and Bower (2016) have critically appraised the methodology and data analysis described in this paper. I am therefore not going to repeat what has already been expertly written, focussing instead on the patient care and field worker practicalities of this tool.

 Involvement from the field is raised on several occasions in the article, however it remains unclear to what lengths the designers and implementers used the returnees’ knowledge on the field realities to make this tool suitable for the task in-hand, beyond “informal discussions”. All volunteers returning from Ebola missions were routinely interviewed in the European headquarters by the organisation, to what extent this was used as an opportunity by the developing team is unclear, if not utilised was this a missed opportunity that could be added to their list of “lessons identified”. It would be useful to know how many returnees were interviewed, the style of interview used and how the information gained guided the designers. Furthermore, given that all returnees were debriefed at head office it would be worth stating if the returnees were all systematically questioned in a standardised manner, or if specific persons were chosen and how this choice was made.

 The field staff who used both the alpha version and full prototype were also interviewed, it would be informative to the reader to understand how many staff were involved in these interviews, whether they were expatriate or local staff and what their specific job role was. The experience of a European doctor may be different to that of a local nurse or health care assistant.

 The authors rightly discuss the difficulty of working in the full Personal Protective Equipment (PPE) that is necessary to remain safe inside the “high risk” area of the Ebola Management Centre (EMC), it would be interesting to gain a deeper insight into how much this was factored into the overall program design. For example, visual difficulties and clumsiness were often described as part of the challenge of working in PPE. Were large buttons or colour coding on the tablet part of the design to assist the healthcare worker overcome these difficulties?

 During very busy times at the peak of the outbreak a numbered system was used to quickly identify the patients with the most immediate needs, a score from 1 (very well) to 5 (critically ill) was assigned to each patient. It would have been informative to know if this or other field-designed approaches were considered or incorporated into the program. Whilst the focus of the tablet appeared to be for transfer of medical information, there is much communication that must also be made between the EMC workers. Identification and location of deceased patients must be efficiently passed to hygiene teams, or safety concerns to logistical teams, was inter-disciplinary communication considered as part of this program?

 Similarly, whilst communication on patient symptomatology was traditionally shouted across the fence and then transcribed into folders outside the “high-risk” area, treatment decisions also needed to be remembered or shouted over. It is not entirely clear how much the tablet was utilised for recording treatments given, for example intravenous fluid administration, hygiene procedures or palliative care medications. This would be valuable from a patient care perspective and likely reduce prescribing errors, missing or duplication of doses. Retrospectively this could also have added to data on what treatment and care was being given and how often. Commonly a generic recipe of medications was administered to all patients from admission into the EMC, this could have therefore been pre-programmed into the tablets as an electronic prescription.

 The method of shouting information across the fence in the EMC, whilst this was often a part of high to low risk communication, was not exclusive. Digital photography that could later be transcribed was often used, particularly during times of high activity or when transferring patients from one location to another. Similarly, only those patients that were bedbound or too unwell to speak with healthcare workers from across the fence required examination and questioning in full PPE. Hence for many inpatient interactions the “high-risk” tablet would not be required. Explanation of how these other interactions were incorporated into the data gathering would be useful, particularly for the completeness of the information generated. The benefits of being able to talk with a patient across the fence, where the clinician was recognisable (no PPE needed) and there were no time constraints due to heat stress, meant that this was a desirable way of working whenever possible. However, it would not negate the use of the tablet as information could still be recorded on the hand-held device.

 The stated aim of making the most effective use of the limited time in PPE to treat patients has not been quantified. Did the tablet assist the healthcare worker in treating the patients, did the number of patients receiving clinical review or treatment change as a result of the tablet or were the health workers able to achieve the same quality of work in a shorter period of time? I think these would have been important questions to ask, rather than the focus being on the ease of data gathering and transfer, it could have been on quality and availability of patient care, perhaps including patient outcomes.

 I found the concept of using radio-frequency identification tags particularly interesting, this would have had great value both for finding and identifying patients for examination, but also importantly for identification and tracking of the deceased. Perhaps because this intervention came during the tail-end of the outbreak there is little discussion of how the tablet could be used for the management of deceased patients, though this was a major issue particularly during the peak of the outbreak when there was a high mortality rate. Unfortunately, the radio-frequency tag aspect of this project did not come to fruition, however a discussion on how this could be moved forward would be worthwhile.

 The potential benefits in the efficiency and quality of data collection gained through the use of the electronic tablet system are clear. How this translated into improving patient care seems less apparent. The balance between patient benefit and data for the sake of research and publication remained controversial throughout the Ebola outbreak. Whilst I don’t question the motives of the team behind this innovation, it would be interesting to know how they managed the balance of this innovation being primarily for patient rather than organisational benefit.

 The honesty of the authors and implementing team is to be applauded, though deeper discussion on how to move forward would have been of value. This venture came at a high price, and though it was not the success they had hoped for it is certainly not an entirely failed project either. Whilst the authors have identified lessons, it is not clear how they have been learnt. The conclusion would benefit a wider discussion of what next, where this technology can continue to be of use and developed to meet its ultimate aim: saving lives.

 The technology is presented as being developed for the specific situation of the EMC, however it would be easily transferable to many humanitarian settings where large numbers of patients or biohazard risks limit the accuracy and efficiency of traditional clinical record keeping. Measles, yellow fever, hepatitis E, cholera and Lassa fever outbreaks would all be well suited to the continued use and development of a potentially very useful field tool. The authors do refer to their vision of the future, and the plan to test a modifiable version of this technology in a nutrition project, a greater emphasis on this vison and how the lessons identified will be learned for the future could have been of use.

 As previously stated, humanitarian technology is a rapidly growing and developing phenomenon. The challenges, lessons and future plans identified by the authors are a useful reminder of how best intentions can go askew without the correct planning and inter-agency communication. These lessons are unlikely be unique to this situation, being broadly adaptable. The honesty of the authors on the difficulties in collaborating with Google is commendable, the next step though is who and how they will work with next. MSF has an opportunity to work within and alongside others in the humanitarian technology field, perhaps partners with the experience to assist them in avoiding these common pitfalls again.

I have read this submission. I believe that I have an appropriate level of expertise to confirm that it is of an acceptable scientific standard.

# Response

**Published:** 2016-09-22

**Authors:** kiran jobanputra

**Affiliations:** Médecins sans Frontières , UK

The authors would like to thank the reviewer for their thorough and constructive critique of this manuscript. We have organised our response by theme:

**User involvement: **extensive user consultation was carried out, as well as user testing with national staff, international staff returning from the field and clinicians who had worked in comparable settings. Several design features addressed the challenges of working in PPE, including large buttons. We have clarified this in the text.

**RFID**: Rapid identification of patients, including the sickest patients, was originally part of the original scope of the project; RFID identification would have helped address this. This was however dropped from the scope as the patient numbers declined and the priority shifted towards transfer of patient data outside the high risk area.

**Treatment module: **a module to record treatments administered was planned and worked on, but not completed and put on hold when it was clear that the outbreak was reducing in scale and thus the opportunity to field test the tablet was highly time sensitive.

**Using these tools for research:** The impetus to develop the EMR was to support improved patient care, including through more efficient use of staff time within the high-risk zone. There were no plans to use the tablet-based EMR for research, although this would be a potential application of this technology.

# Peer review: Referee response for version 1

**DOI:** 10.5256/f1000research.8913.r14552

**Published:** 2016-07-11

**Authors:** James Whitworth, Hilary Bower

**Affiliations:** 1 Department of Infectious Disease Epidemiology, Faculty of Epidemiology and Population Health, London School of Hygiene and Tropical Medicine, London, UK; 2 Department of Infectious Disease Epidemiology, Faculty of Epidemiology and Population Health, London School of Hygiene & Tropical Medicine, London, UK

The authors present a honest evaluation of an operational research project to innovate a new tool for recording, visualising and sharing medical data in response to the challenging needs of a high risk infectious situation in a humanitarian emergency, namely the outbreak of Ebola in West Africa 2014-15.  This is an interesting and valuable paper about developing a clinical information and patient management system based on tablet computers that can be used in Ebola Treatment Centres and similar high risk environments. It is mainly a narrative paper with little in the way of quantitative results. The lessons learned are fairly generic to any project management challenge, and go beyond those of innovation in humanitarian settings.

 They describe the process of developing a tablet and new software to respond to the complex requirements of high bio-hazard infection control, and compare this tool with standard practice in this setting. They note the urgency of the project, which was intended to improve clinical care, and by implication outcomes, for patients by helping medical staff make more efficient use of the limited time they could spend in the red zone due to the constraints of personal protective equipment.

 The title is appropriate and the abstract is generally clear, although I felt that the description of the comparison of tablet and paper-based data was not clear. It was not just tablet data that were analysed, and what is meant by ‘record “pairs”’ is not clear just from reading the abstract.

 The background and methods are mostly clear and appropriate. I am not an expert on computer hardware or software, so cannot comment on the appropriateness of the systems selected. The researchers used a mixed methods approach appropriate for the different aspects of the project they wished to evaluate: namely need identification, buy-in, implementation, tool functionality and maintenance, and a measurement of data quality. They have identified key issues that delayed the process of development and the limited testing of the tool they were able to achieve.

 I am astonished at the cost of development of the system, which seems to have been driven largely by the salary costs and person-hours involved, as well as producing customised hardware.

 It was not clear to me quite how the data collected in the high risk zone were exported for further analysis and storage. Was this by disinfecting the tablets in chlorine and physically removing them from the high risk zone, or was it through connection some form of local area network that allowed the data collected in the high risk zone to be electronically transmitted outside? This could be made clearer.

 I wondered about issues of confidentiality of patient data being transferred from Sierra Leone to the MSF headquarters in Europe (panel 2). How were these addressed, approved and overseen?

 I was surprised that no data were collected on clinical management according to panel 3. This seems like a missed opportunity, as further research into optimal management of cases of ebola is still required. There is comment about this at the end of the discussion (page7), but was this an oversight when the system was being developed?

 Did this study undergo any ethical scrutiny in Sierra Leone? I would imagine that it should have done, but this is not mentioned.

 Methods para 6:

- Suggest rephrasing the second sentence to make it clear that this part of the paragraph refers to data extraction for the tablet, and the latter to the paper/excel database.
- Is it a correct understanding that only the first tablet entry of the day was taken into account? If so, could you mention why it was not possible to take account of subsequent entries given the relatively small number of records under analysis
- Did practitioners entering data in the high risk zone review their own entries once out? Our question refers to the comment in the first para of the discussion about opportunity for correction. It might be useful to include a paragraph on the procedure for using the tablet.

 

 The results are very interesting, and it is good to see an honest description of the problems encountered with this project. Their results highlight areas of tension in the process and detail the successes (a functioning tool with good data capture) and failures (implementation too late to have impact on patient care, and lacking one of the initial requirement – a way to find patients in a large facility) of the project. From both they draw out lessons for future innovation between humanitarian and technological enterprises.  They also detail the technical specifications and overview costings for the tool.

 I was not clear what was meant by ‘transfer of knowledge of the hardware from Google to MSF did not occur’ (page 5). Further on in the discussion section of the paper there is a comment about the transfer of knowledge, but this appears to be related to the software rather than the hardware. Indeed, it seems that this problem has been overcome and the software has been adapted for use in other emergencies (page 6).

 I would have appreciated more information about the ‘significant work needed to clean and analyse the exported data’ from the tablet. Why was that necessary?

 The results are generally clearly described, and suggest that the tablet may be at least as good as paper records, though the analysis currently does not appear to take into account the role of chance, and a proportion of records (17% ?) had to be excluded. The qualitative study is quite weak. It appears that different data were collected systematically using the two different systems, so only some of the data were collected using both methods.  Further comments on the quantitative study are:

- If we understand correctly the agreement between the two methods was calculated by simple percentage agreement: would it be possible to perform a Kappa test to take into account chance?
- What was the range of interaction pairs per patient?  Was any difference seen in the recording by either method related to the severity of the patient’s illness, or the number of people admitted at the time?
- Is it possible to state the percentage of missing data overall and/or by recording method and comment on the implications for the agreement results? On rough calculation overall it seems to be 17% (204-(85x2) = 34, 34/204) missing data in one or other system.

 

 The agreement between data sources ranged between 69 and 95%. This is rated as ‘high consistency’ but I was not sure on what basis. It was not clear to me, which collection method was felt to be the gold standard, or even if this was determined before the comparison. I sense from the discussion, the tablet was thought to perform better than the paper method, but I am not convinced about this from the results.

 There was clearly a tension between MSF wanting a ‘good-enough‘ product, and Google being perfection-driven. I suspect this was not just a question of communication, there is likely to be some underlying differences in corporate culture, which might be hard to bridge even with all the time in the world.

 What is meant by ’Google’s haste to move onto the next project’ (page 6). Was this ebola-related, or simply reflecting that this department at Google was busy with a range of different projects?

 The discussion was well written and interesting. The authors claim that this is the first such device to be developed. How did they search for other evidence of similar projects? I heard anecdotally of other projects, but am not aware whether they reached fruition, or have ever been published in any form. It would be good to have a thorough systematic search to establish what else has been developed.

 Is it correct to assume that the more frequent and detailed data available with the tablet related to the recording of vital signs, which were not entered from the paper record at the time of analysis?

 Could you comment on the value gained from the additional encounters recorded in the tablet and whether users found recording real-time information was clinically useful? 

 It is not clear to what extent this system can be made available to others. The reference to the consortium being established is to a website that is being prepared.

 What data are available from MSF in Amsterdam? Are they data related to the development of this tablet computer based system or the patient data from this Ebola Treatment Centre? This is not clear to me.

 In our view, however, the authors present a well-targeted piece of operational research undertaken in challenging circumstances with transparent insights into where the development process fell short. They make it clear that their results are limited, but provide a foundation for moving forward.

We have read this submission. We believe that we have an appropriate level of expertise to confirm that it is of an acceptable scientific standard, however we have significant reservations, as outlined above.

# Response

**Published:** 2016-09-22

**Authors:** kiran jobanputra

**Affiliations:** Médecins sans Frontières , UK

The authors would like to thank the reviewer for their thorough and constructive critique of this manuscript. We have organised our response by theme:

**Record pairs:** All interactions continued to be captured on paper during the pilot of the tablet-based EMR, and the comparison included all those clinical entries that had been captured by both means (referred to as “record pairs’.

**Data transfer: **Data was transferred wirelessly to a server outside the high risk zone via a secure local area network, which indeed was one of the key advantages of this technology for Ebola Management Centres.

**Confidentiality**: Tablet data included only an ID code and approximate date of birth based on current age, so although data was linked to an individual, the individual was not identifiable.

**Treatment module: **A module to record treatments administered was planned and worked on, but not completed and put on hold when it was clear that the outbreak was reducing in scale and thus the opportunity to field test the tablet was highly time sensitive.

**National oversight and ethics approval**: This was not a study as such, but an ad hoc evaluation of an operational innovation that was introduced at the peak of the emergency. The evaluation had no impact on patient experience, since it did not involve subjecting patients to any process additional to that which they would already be undergoing; no additional patient data was collected for this evaluation. Interviews with staff were informal and focused on experience of using the tablets. As such, it met the MSF ERB criteria for exemption from formal ethics review and was approved by the MSF medical director. The tablet-based EMR was discussed with and demonstrated to health authorities prior to implementation, who were supportive of this innovation and did not suggest additional ethical scrutiny.

**Data checking and validation: **Practitioners were able to review the data at any time, including within the high-risk zone, so were able to check for consistency. Since the implementation team regularly checked for consistency between data entered into the tablet, and data on the server, it was not deemed necessary to ask the clinicians to do this themselves.

**Why was knowledge transfer not successful?:** Due to the hasty decommissioning of the team, there was insufficient time for a comprehensive handover (from Google to MSF) of information and understanding required to operate and maintain the software and hardware that had been developed. In the case of the software and key hardware, MSF was able to hire an ex-Google engineer who worked on this project to further develop the software and complete the process of handover to MSF. However some parts of the hardware (e.g. polycarbonate casing) were deemed too expensive to warrant further investment by MSF, so little attempt was made to obtain the knowledge necessary to produce these.

**What was the 'significant work needed to clean the data'? **The database was not configured to create a single record for a patient encounter if the user moved in and out of the record, resulting in upto five partial records within a 10 minute period which all related to a single encounter, and thus required to be merged into a single record of that encounter. In addition, new users entered a variety of practice data which was retained in the database, and needed to be identified and removed. Finally, records were exported with content and order based on OpenMRS coding, resulting in 3 data fields per data element and an order that was not linked to the logical grouping and order on the tablet forms.

**How reliable was the comparison of tablet and paper data?: **Of 204 tablet records, 119 did not have an equivalent record in the Excel database because only a single set of observations were recorded each day on paper whereas on the tablet additional observations were sometimes recorded later the same day. Therefore only one record pair per day was possible. Of 111 encounters recorded on paper, 28 were not matched to a tablet record.

 The median number of record pairs per patient was 2.0, with a maximum of 19 for a patient who was admitted for 19 days. There were relatively few patients admitted during the implementation, many of whom were later confirmed as not having Ebola, so while the influence of severity of illness or patient load on the data quality are interesting in theory, it cannot be validly assessed.   

**To what extent can this system be made available to others? **The software is available on-line and can be down-loaded for free; the code is also freely available for developers to use. Several of the developers involved have formed an open-source community that is supporting this code, which has replaced the consortium in this regard.
